# Inflammatory Myofibroblastic Tumor of the Nasal Septum

**DOI:** 10.1155/2013/670105

**Published:** 2013-07-10

**Authors:** Yuri Okumura, Kazuhiro Nomura, Takeshi Oshima, Atsuko Kasajima, Takahiro Suzuki, Eichi Ishida, Toshimitsu Kobayashi

**Affiliations:** ^1^Department of Otolaryngology-Head and Neck Surgery, Tohoku University Graduate School of Medicine, 1-1 Seiryo-cho, Aoba-ku, Sendai, Miyagi 980-8574, Japan; ^2^Department of Pathology, Tohoku University Hospital, 1-1 Seiryo-cho, Aoba-ku, Sendai, Miyagi 980-8574, Japan

## Abstract

We report an extremely rare case of inflammatory myofibroblastic tumor of the posterior edge of the nasal septum. An 11-year-old boy presented with frequent epistaxis and nasal obstruction persisting for one year. Based on the clinical presentation and imaging studies, juvenile angiofibroma was suspected, but angiography suggested the possibility of another type of tumor. Transnasal endoscopic surgery found that the tumor protruded into the nasopharynx from the posterior end of the nasal septum. Histological examination identified spindle cells with immunoreaction for vimentin, smooth muscle actin, and anaplastic lymphoma kinase (ALK), but not for desmin and cytokeratin. This is a report of inflammatory myofibroblastic tumor mimicking juvenile angiofibroma. This case suggests that angiography is helpful in the differential diagnosis of epipharyngeal tumor in adolescence.

## 1. Introduction

Inflammatory myofibroblastic tumor (IMT) was first observed in the lung in 1990 [1], but extrapulmonary IMTs have also since been reported. IMT is classified as myofibroblastic neoplasm with intermediate grade, which rarely metastasizes. The clinical presentation is a neoplastic process with recurrence and metastasis in some cases [2, 3]. IMT may occur throughout the body and is most commonly found in the lung, abdominal cavity, retroperitoneum, and extremities but is uncommon in the head and neck region [1, 4]. We present a case of IMT located in the nasopharynx, which mimicked juvenile angiofibroma.

## 2. Case Presentation

An 11-year-old boy had visited a provincial hospital because of frequent epistaxis and nasal obstruction persisting for one year. A tumorous mass was found in the nasopharynx, so computed tomography (CT) and magnetic resonance (MR) imaging studies were performed. Juvenile angiofibroma was suspected, so he was referred to our hospital for further examination and treatment.

**Figure 1:**
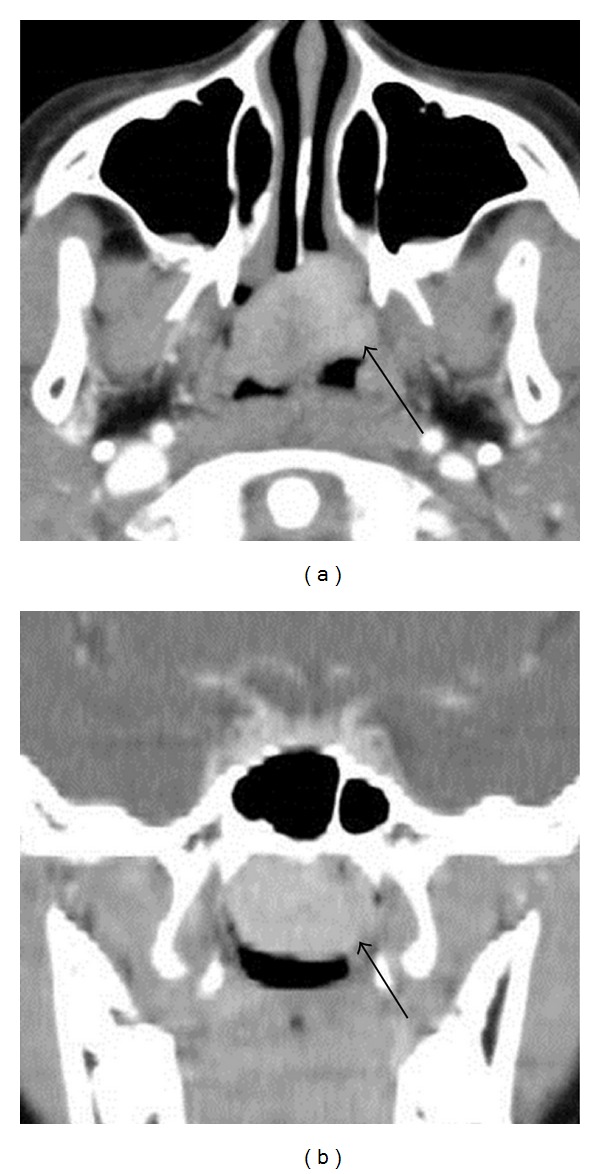
Axial (a) and coronal (b) computed tomography scans with contrast medium showing a soft tissue mass in the nasopharynx with homogeneous enhancement (arrows).

**Figure 2:**
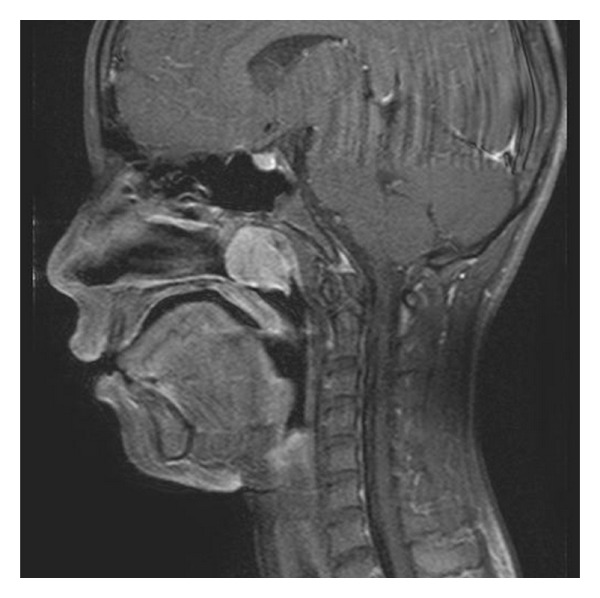
Sagittal T1-weighted magnetic resonance image with gadolinium showing a soft tissue mass with homogeneous enhancement.

**Figure 3:**
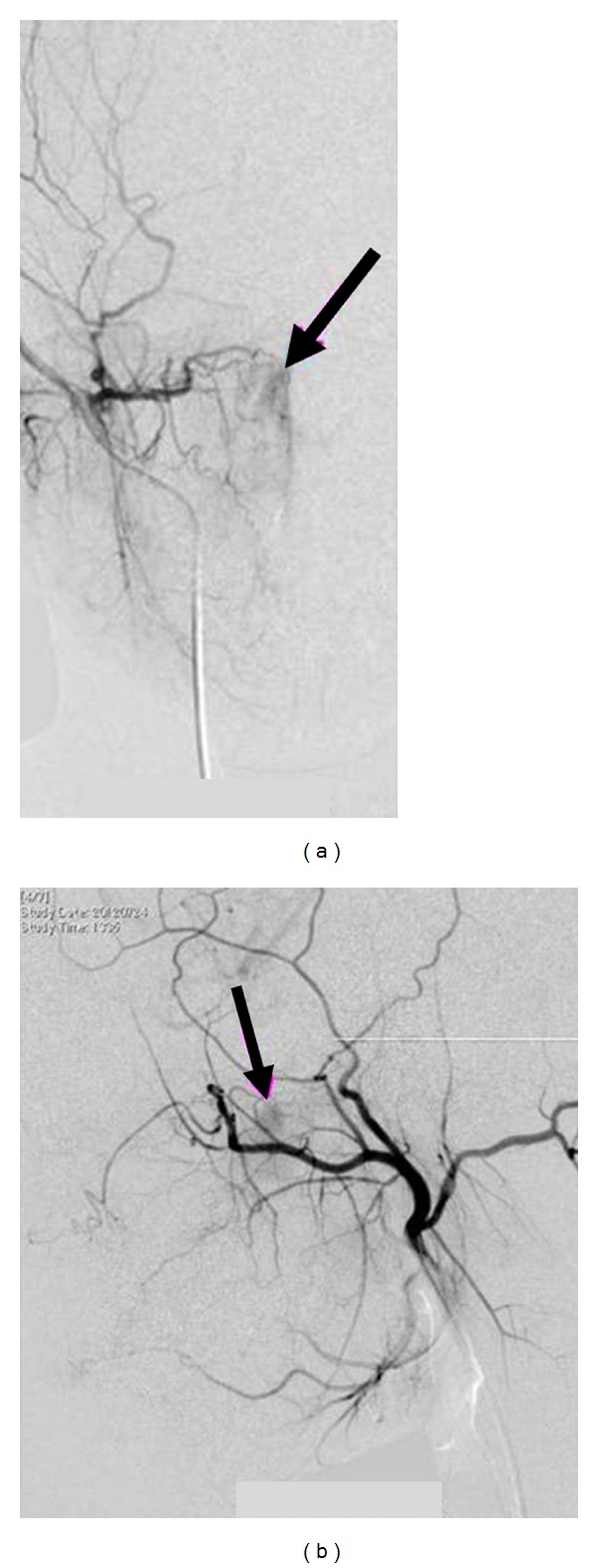
Frontal (a) and lateral (b) right external carotid angiograms showing faint tumor staining (arrows).

Physical examination found a smooth reddish mass in the nasopharynx. The imaging studies performed at the previous hospital were reexamined. CT scans showed a homogeneously enhanced soft tissue mass in the nasopharynx without bone destruction (Figure 1). The T1-weighted MR image with contrast medium demonstrated an isointense mass with homogeneous enhancement (Figure 2). These findings elucidated the diagnostic impression of juvenile angiofibroma, as suggested by the previous physicians. Angiography detected faint tumor staining, but no obvious feeding artery (Figure 3). This result suggested the possibility of tumors other than juvenile angiofibroma because of the unexpectedly poor vascularity. Histological examination of the specimen obtained by transnasal biopsy was highly suspicious of malignant neoplasm, such as follicular dendritic cell sarcoma, malignant melanoma, or mesenchymal tumor because of the high cellularity, large nuclei, and prominent nucleoli.

Transnasal endoscopic extirpation of the tumor was performed under general anesthesia one week after the biopsy. The tumor was a red mass with a smooth surface originating from the posterior end of the nasal septum. The tumor was totally removed en bloc by resection at the posterior edge of the nasal septum. The surgery was completed with little blood loss and no complications.

**Figure 4:**
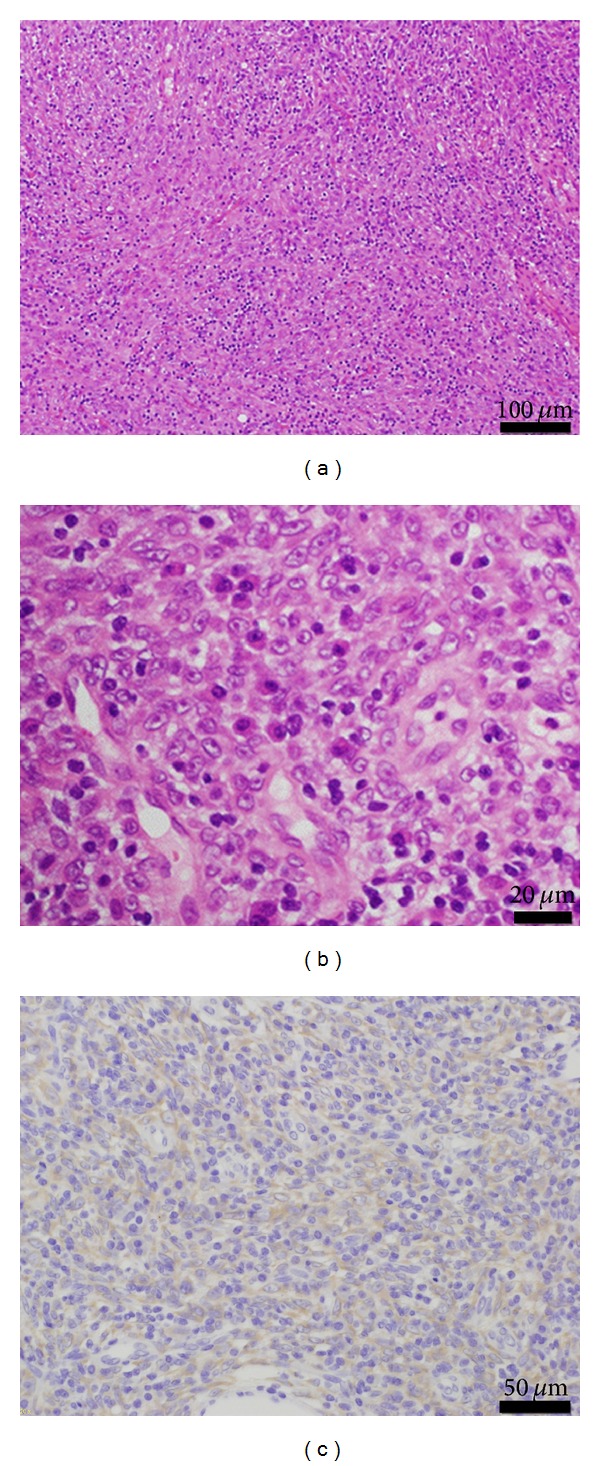
Photomicrograph of the tumor specimen showing spindle cells with infiltration of lymphocytes, plasma cells, and foamy histiocytes ((a), (b): hematoxylin and eosin staining). Immunohistochemical staining showing diffuse cytoplasmic reactivity for ALK (c).

Histological examination with routine hematoxylin-eosin staining demonstrated proliferation of spindle cells with enlarged nuclei and infiltration of lymphoplasmacytic cells (Figures 4(a) and 4(b)). Mitotic figures were rarely detected, and no necrosis was identified. Immunoreactivity for vimentin and smooth muscle actin (SMA) was detected in the cytoplasm of the tumor cell, but not for desmin or cytokeratin. This immunoprofile revealed myofibroblastic differentiation of this tumor. Additionally, anaplastic lymphoma kinase (ALK) was weakly expressed in the tumor cells (Figure 4(c)). These findings confirmed the final diagnosis of IMT.

The patient showed no evidence of recurrence at 8 months after surgery.

## 3. Discussion

IMT manifests as various symptoms depending on the site of origin. IMTs in the head and neck region do not always cause systemic symptoms, such as fever, weight loss, and pain, unlike IMTs in visceral organs [5]. IMTs may occur throughout life with no sex difference [4]. IMT of the nasal cavity is rare, and the principal symptoms are nasal obstruction, epistaxis, and nasal discharge. The mass is covered by normal or edematous mucosa, so it is easily misdiagnosed as a nasal polyp in the early stage [4].

IMT has no characteristic findings on CT or MR imaging, but invasive extension to the surrounding tissues may lead to misdiagnosis of malignant neoplasms [6, 7]. Findings may be similar to those of juvenile angiofibroma. Superselective embolization was performed preoperatively because of the hypervascular nature of the tumor and mildly hypertrophic feeding arteries [4], but no other reports about angiography of nasal IMT are available.

In our case, angiography showed only faint staining of the tumor and no obvious feeding artery. Although the clinical presentation and imaging studies were confusingly similar to juvenile angiofibroma, the angiography findings suggested the possibility of another type of tumor, so we decided to perform biopsy preoperatively.

Pathological examination is essential to diagnose IMT. Histologically, IMT consists of myofibroblastic spindle cells with prominent infiltration of lymphocytes and plasma cells. Three histological patterns have been proposed [2]: (1) myxoid, vascular, and inflammatory areas resembling nodular fasciitis; (2) compact spindle cells with intermingled inflammatory cells (lymphocytes, plasma cells, and eosinophils) resembling fibrous histiocytoma; and (3) dense plate-like collagen resembling a desmoid or scar. In most cases, individual tumors contain various proportions of these patterns. In our case, the tumor consisted mostly of the second pattern of diagnostic criteria but also partly of the other patterns.

Immunohistochemically, vimentin has been expressed most frequently (99%) in extrapulmonary IMTs, as well as SMA (95.8%), desmin (69%), and cytokeratin (36%) [2]. In our case, the tumor cells were positive for vimentin and SMA and negative for desmin and cytokeratin. Based on these findings, myofibroblastic differentiation of the tumor cells was confirmed and the tumor was diagnosed as IMT.

Recently, ALK gene rearrangements have been reported in 50% to 75% of extrapulmonary IMTs [8], which supports the neoplasmic nature of IMT. ALK is a receptor tyrosine kinase and is expressed in the majority of anaplastic large-cell lymphomas (ALCL). ALK-positive ALCL is associated with younger age and better prognosis, but there is no clear relationship between ALK expression and response to therapy in IMTs. According to previous studies, ALK-positive IMTs are diagnosed at a younger age and have a higher recurrence rate, and ALK-negative IMTs correlate with the presence of metastasis [8].

Corticosteroid administration, radiotherapy, chemotherapy, and surgery have been used as treatment modalities for IMT. The definitive therapy of extrapulmonary IMTs, including head and neck IMTs, seems to be total surgical excision, which is curative in more than 90% of cases [2, 6]. On the other hand, the local recurrence rate of extrapulmonary IMT may be as high as 25%. The metastatic rate of IMT is low, ranging from <5% to 11% in different series [7]. Some recurrences have been reported in cases of nasal IMTs [6, 9], but no metastasis has been described in the English literature.

The present case of IMT of the nasal septum shows that, to avoid unnecessary overtreatment, the correct diagnosis must be established based on histological and immunohistochemical examinations. Although the tumor was thought to be removed completely, close followup is essential because the tumor was ALK positive, and recurrence in months to years after the surgery has been reported [1, 2].
